# Double trouble

**DOI:** 10.1186/2041-2223-4-12

**Published:** 2013-06-27

**Authors:** Mark A Jobling

**Affiliations:** 1 Department of Genetics, University of Leicester, University Road, Leicester LE1 7RH, UK

## 

“How unique are you?” – an infuriating question for anyone with a pedant’s ear for linguistic correctness, but one that’s posed many a time in the fervid world of public engagement in genetics. And the expected answer is ‘very’ - pedants notwithstanding. The conventional way to demonstrate this is to ask a series of questions about traits with a supposedly simple genetic basis: tongue-rolling, hitch-hiker’s thumb, direction of hair whorl, cleft chin, attached earlobes… Often a tiny square of innocent-looking paper is proffered, with instructions to place it on the tongue (rolling or otherwise); to some people it’s tasteless, while to others it’s bitter due to its content of phenylthiocarbamide, meriting a sugary antidote in the form of a Polo mint. Sometimes there’s a vase of freesias whose scent fills the air – at least, for those of us whose genes allow us to detect it. Less savoury aspects involve interrogations about the colour or the smell of a subject’s urine following the eating of beetroot or aspgus.

Testing many such traits in a large group demonstrates that few people share the same combination, and emphasises how genetically different we all are. The problem is that, as John H. McDonald’s splendid *Myths of Human Genetics* website [1] illustrates, many of these traits are continuous rather than discrete, and most do not have a simple genetic basis at all. Tongue-rolling, for example, certainly has some genetic component, with children of tongue-rolling parents more likely to be rollers themselves, but is not a simple dominant phenotype, as is often assumed. Hitch-hiker’s thumb, cleft chin, and attached earlobes are assumed in the ‘human uniqueness’ tests to be binary characteristics, but in fact all show continuous variation.

Apart from family studies, another way to investigate the genetic components of traits is to compare concordance in twins. It was Francis Galton who realised their possible value ‘to weigh in just scales the effects of Nature and Nurture’ [2]. He sent questionnaires to twins and their relatives, and having built a respectable database was interested to find that ‘similarity and extreme dissimilarity between twins of the same sex are nearly as common as moderate resemblance’. We now refer to the twins who are so alike that ‘the one is sometimes fed, physicked, and whipped by mistake for the other’ as monozygotic (MZ – ‘identical’), since they arise from the division of the same zygote, rather than dizygotic (DZ – ‘fraternal’). MZ twins are certainly very similar, but not identical - traits such as hair whorl, tongue rolling, and the effect of a bowl of borscht on the urine all reveal cases where one twin differs from the other [1].

A very simple question, as posed by the Swedish twin registry in 1961 [3] sorts out the MZ/DZ issue in 95% of cases: “During childhood, were you and your twin as alike as two peas in a pod, or not more alike than siblings in general?” (to be precise, the Swedes actually asked whether twins were ‘similar as two berries’). With the arrival of DNA fingerprinting, and its descendant technology STR profiling, it became a matter of science to distinguish between twin types. Indeed, in the 1990s Alec Jeffreys analysed the DNA of 11-year-old twin sisters for a popular BBC television programme, “Jim'll Fix It” (fronted by the now deceased and disgraced Jimmy Saville). The DNA fingerprints, set up behind a curtain, were revealed to be identical, confirming what anyone could tell by the simple double-take of looking at the girls.

The indistinguishable DNA profiles of MZ twins can pose a problem in forensic analysis. One in 300 people has such a twin, and if they leave a DNA sample at a crime scene and other evidence fails to exclude their sibling, then how do we know which is responsible? Naturally enough, witness statements can be very unreliable. In one of Galton’s case studies (again featuring corporal punishment): ‘Two twins were fond of playing tricks, and complaints were frequently made; but the boys would never own which was the guilty one, and the complainants were never certain which of the two he was. One headmaster used to say he would never flog the innocent for the guilty, and another used to flog both’.

There have been a number of real cases in which this difficulty has arisen. One comes from November 1999, when a young woman was raped in the town of Grand Rapids, Michigan; the crime was unsolved until 2004, when Jerome Cooper, in jail at the time for a sex offence, applied for parole, requiring him to provide a DNA sample. His profile was checked against a list of unsolved crimes, and matched DNA recovered from the 1999 rape. Case solved? – not quite, because of the existence of Jerome’s MZ twin brother, Tyrone, who was also in the area at the time of the rape. The practice of the first headmaster had to be followed – it was impossible to jail both brothers for the crime, so they remained free. Similar cases involved putative jewellery thief Hassan or Abbas O. in Germany, and putative rapist Darrin or Damien Fernandez in Boston.

A more recent French case has been widely reported: police investigating a series of sexual assaults in Marseille arrested a 24-year-old unemployed delivery driver, but the arrestee had a twin brother (with the same non-profession), and a victim could not tell them apart. The DNA left at the crime scenes was of no use, at least as far as a conventional DNA profile was concerned. News reports [4] suggested that an ‘ultra-sophisticated genetic test’, might help, and gave the price-tag of this mysterious product as 1 million euros ($1.3 M).

This seems a lot of money – what test can they possibly mean? Differences in copy-number of DNA sequences [5], or in epigenetic modifications such as DNA methylation [6] between MZ twins in matched tissues have been reported, and might in principle help, but neither would be easy to get past a skeptical defence lawyer. As next-generation DNA sequencing continues to fall in cost and rise in power, it’s there we need to look. The tissue of interest in the French case, it’s safe to assume, is sperm. Could we find simple sequence differences between the genomes of the sperm populations of each twin, which had arisen during the development of either of them from the original zygote from which they both derived?

The zygote splits after fertilisation to give two identical single-celled proto-twins, which then undergo on average about seven cell divisions in the first week of development to give embryos each consisting of about 100 cells. In the subsequent week the primordial germ cells, which give rise to the stem cell population of spermatogenesis in each male, become detectable. So let’s take 2 × 7 as a conservative minimum number of cell divisions that clearly septe the primordial stem cell populations of each twin. Any heterozygous somatic mutations that arose during these 14 cell divisions are expected to be present in half the sperm of one twin, but absent from the other. If we carry out high-coverage whole genome sequencing from a donated sperm sample of each twin, with a mutation rate of 0.05 × 10^-9^ per nucleotide per cell division [7], and a diploid genome size of 6 × 10^9^ bp, this back-of-an-envelope calculation suggests that we expect to find ~4 such differences between the sperm samples (there will obviously be other, later-occurring variants that exist at lower frequencies). Having identified the variants, they could be confirmed by conventional DNA sequencing, and then typed in the crime-scene samples. Four nucleotide differences seems alarmingly few, but at only ~$6000 per high-coverage genome sequence [8] it seems worth a try.

All the same, it’s a pity that a simpler and better-established forensic tool was not available in this case, which can answer the “how unique are you?” question in a pedant-satisfying way. The inquisitive Galton [9] noted that the friction ridges on our fingertips (actual, rather than DNA fingerprints), while more similar between twins than non-twins, are nonetheless different enough to allow them to be distinguished every time.
